# Therapeutic Application of Lithium in Bipolar Disorders: A Brief Review

**DOI:** 10.7759/cureus.29332

**Published:** 2022-09-19

**Authors:** Zubair Mahmood Kamal, Siddhartha Dutta, Sayeeda Rahman, Ayukafangha Etando, Emran Hasan, Sayeda Nazmun Nahar, Wan Farizatul Shima Wan Ahmad Fakuradzi, Susmita Sinha, Mainul Haque, Rahnuma Ahmad

**Affiliations:** 1 Psychiatry, National Institute of Mental Health (NIMH), Dhaka, BGD; 2 Department of Pharmacology, All India Institute of Medical Sciences, Rajkot, Rajkot, IND; 3 School of Medicine, American University of Integrative Sciences, Bridgetown, BRB; 4 Department of Medical Laboratory Sciences, Faculty of Health Sciences, Eswatini Medical Christian University, Mbabane, SWZ; 5 Public Health, Institute of Epidemiology Disease Control and Research (IEDCR), Dhaka, BGD; 6 Public Health, Save the Children Bangladesh, Dhaka, BGD; 7 Community Medicine, Faculty of Medicine and Defence Health, National Defence University of Malaysia, Kuala Lumpur, MYS; 8 Physiology, Khulna City Medical College and Hospital, Khulna, BGD; 9 Pharmacology and Therapeutics, National Defence University of Malaysia, Kuala Lumpur, MYS; 10 Department of Physiology, Medical College for Women and Hospital, Dhaka, BGD

**Keywords:** nhs approved medications, fda-approved medications, treatment modalities, history of bipolar disorders, bipolar spectrum disorder, narrative review, bipolar disorders, lithium, intervention, pharmacological treatment

## Abstract

Bipolar affective disorder includes Bipolar Disease (BD) and Bipolar Spectrum Disorder (BSD). The prevalence of BSD, BD-I, BD-II, and subthreshold BD globally is estimated to be about 3.1%, 1.5%,0.03%, and 1.6%, respectively. BD is a multidimensional disease that exhibits a range of moods of mania, hypomania, and depression. The disease is chronic, complex, and fatal, with a high possibility of reappearance, infirmity, social incompetence, and felo-de-se. Managing emotional disruption, negative neuropsychology, physiology, and immunology is a challenge. This review focuses on therapeutic benefits, adverse drug reactions, and pharmacological intervention for BD and BSD, in particular lithium. Long-term management of BD with a single medication is ineffective and therefore, not recommended. It is advised to use multiple agents for treatment instead. Medications include mood stabilizers (lithium and anticonvulsants), atypical antipsychotics, and antidepressants. Along with medication provision, psychotherapy is of great significance for BD patients. The review was conducted on recent available scientific literature through the electronic database like Embase, ScienceDirect, Google Scholar, and PubMed using keywords like ‘Bipolar Disease,’ ‘Bipolar Disease Therapeutics,’ ‘Bipolar Disease and Psychotherapy’ to highlight the possible effective means of management of this disease of mood instability.

## Introduction and background

Globally, bipolar disorder (BD) and bipolar spectrum disorders (BSD) variants are of jeopardizing psychological diseases [1-3]. Worldwide recent guesstimates of BD lifetime prevalence for BSD, BD-I, BD-II, and sub-threshold BD were 3.1%, 1.5%, 0.03%, and 1.6%, respectively [4]. Multiple studies reported that 50-75% of all cases of BD and BSD live in low-middle-income countries (LMICs) and low-and lower-middle-income countries (LLMICs), and only 10% of them had reasonable healthcare access [3,5]. BD is a multidimensional disease that embraces a range of events (manic, hypomanic, and depressive) of dire emotional disruption. Furthermore, there are serious disturbances in neuropsychology, immunology, and physiology, thereby raising multiple disorders challenging to treat [2,6]. The etiopathogenesis of BD to date is poorly explained. It is frequently described genetic inheritances remain a substantial issue in developing BD [7,8]. It has been revealed that patients with BD and BSD suffer from miserable life patterns [9,10]. The disease affects patients' education, productiveness, performance, socialization, and close and affectionate kinship [9].

Pharmacological interventions remain the principal pillar of treatment options for BD, but drug therapy alone inadequately addresses the issue [11]. Moreover, there is a high possibility of recurrence, enduring symptoms, and psychopathological incapacitation [11]. Psychotherapy is a dynamic component in the overall management of BD [12] and must be available to patients and communities. A few different types of psychotherapy have been recommended and practiced for BD. These are interpersonal and social rhythm therapy (IPSRT), cognitive behavioral therapy (CBT), dialectical behavior therapy (DBT), group psychoeducation, and family-focused therapy (FFT) [11,13]. There are several classes of drugs used to treat BD [14]; among them, lithium (Li) is a classic drug that's used to treat this disease [15]. It's the most effective but possesses some serious toxicity [16].

Objectives of the study

This review focused on the therapeutic benefits and adverse drug reactions of pharmacological intervention of BD and BSD, particularly the therapeutic application of lithium.

## Review

Historical perspective of bipolar disorders

It has been reported that human beings have been experiencing shifting moods and energy levels with a history of over thousands of years [17]. The alternating mood state as highs and lows, termed mania and melancholia, respectively, originated in ancient Greece [17,18]. Melancholia derives from the Greek words melas and chole, meaning "black" and "bile" or "gall," which refers to the compulsive state of severe despondency [19-21]. The word mania originates from the Greek phrase Ania and Manos, meaning "to produce great mental anguish" and "relaxed or lose," respectively [22]. The age-old concept of developing mania was believed as a result of the excessive amount of yellow bile in our system [23]. Additionally, it was presumed in the old days that mania and melancholia arose from imbalances in the body's humor [24-26]. Additionally, older concepts regarding mania and melancholia were because of excess amounts of yellow bile, and black bile, respectively [20,27,28]. A Greek Methodic named Soranus of Ephesus (98-177 CE {common or current era}) describes mania and melancholia as related disorders [29]. Later, Aretaeus of Cappadocia, the most outstanding medical scholar of the 2^nd ^century of Greco-Roman culture, first talked about the BD [30,31].

The contemporary psychiatric comprehension of manic-depressive disease dates back to 1850 [32]. Jean-Pierre Falret (1794-1870), a French psychiatrist, described a novel psychiatric disorder named “folie circulaire” (circular insanity) which follows a cycle of depression, and mania, with a symptom-free interlude of the undetermined period amid these two ends of the disease [21,33-35]. Jules Gabriel François Baillarger (1809-1890), another French neurologist and psychiatrist, described a similar disorder as an alternating phase of mania and melancholia without any remission and termed “la folie à double forme” [36-38]. Emil Wilhelm Georg Magnus Kraepelin (1856-1926), a well-known and persuasive German expert in mental disorders, studied and cataloged the instinctive trajectory of BD and termed it as "manisch-depressives Irresein" (manic-depressive insanity) [39-42]. Dr. Emil Kraepelin is considered a post-Freudian father of modern scientific psychiatry because of his work [39]. He had differentiated dementia praecox (currently known as schizophrenia) and manic-depressive insanity (presently called BD or BSD) as two endogenous psychiatric entities [43,44].

Bipolar disorders and lithium initial findings

Initially, Li^ ^was utilized for the pharmacological management of uric acid calculi and gout. Nevertheless, it was thought to be toxic and ineffective [45]. However, lithium was efficacious for managing psychiatric diseases for over one hundred years [45]. An Australian psychiatrist named John Frederick Joseph Cade AO (1912-1980) had the first detailed positively regarding the pharmacology of lithium carbonate as a mood stabilizer for the therapeutic management of BD in 1948 [46]. Dr. John Cade found that lithium salt effectively controlled manic-depressive episodes of veterans of World War II [45,47]. The fear of lithium toxicity developed quickly, and Dr. Cade’s positive findings were not accepted to be utilized clinically for years [48]. The medical community has accepted Li because Poul Christian Baastrup, Mogens Schou, and their colleagues' relentless work generated evidence of this medication’s efficacy and safety [48-54].

Current pharmacological treatment modalities for bipolar diseases

**Figure 1:**
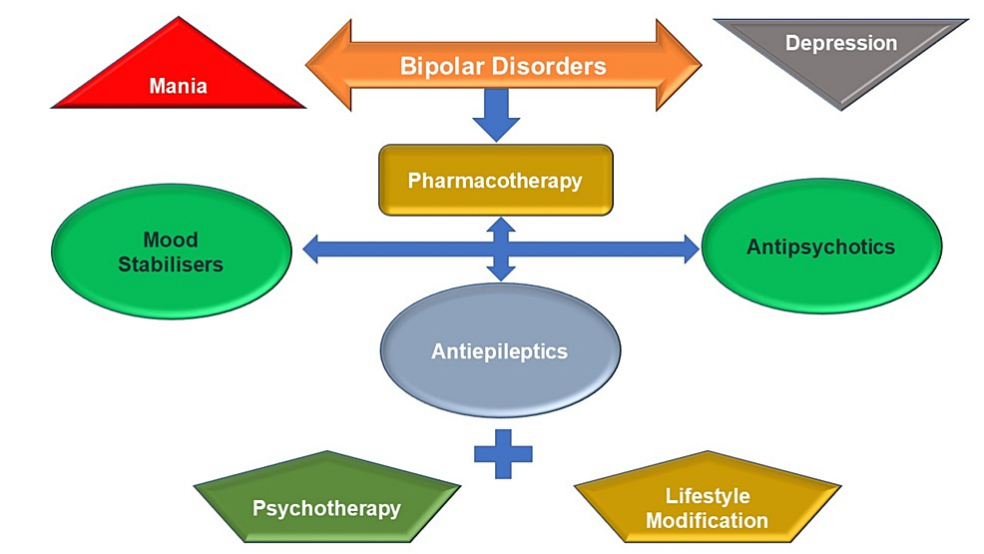
Multimodal Treatment Strategies In Bipolar Disorders. Image Credit: Siddhartha Dutta.

BD is a chronic, complex, and fatal disease with high possibilities of reappearance, infirmity, social amateurishness, and felo-de-se [6,55]. The modalities of treatment are depicted in Figure 1. The pharmacological interventions of BD typically comprise conjunctions of no less than two medications [32,56]. Those medications include mood stabilizers (lithium and anticonvulsants {AC}), atypical antipsychotics, and antidepressants [57-59]. Depressive states are preliminary predominant features and frequently lead to the wrong diagnosis. Thereby, the commencement of mood stabilizing medication is commonly deferred, leading to complicated clinical scenarios [56]. Orthodox mood stabilizing agents are considered for first-line medicines [55]. At the same time, atypical antipsychotics (AP) are gradually increasingly being prescribed [56]. Selective serotonin reuptake inhibitors (SSRIs) are recommended as soon as mood stabilizers are ineffectual and when the depression returns among BD individuals [56].

Table 1 illustrates medications approved by the United States of America (USA) Food and Drug Administration (FDA) [60]. Almost similar medication is approved by the National Health Service (NHS), the United Kingdom (UK) depicted in Table 2 [61].

**Table 1:** FDA Approved Medications for Bipolar Disorder. FDA: Food and Drug Administration

Medication Category	Pharmacological Name and Year Licensed	Manic	Mixed (Mania/ Depression)	Continual Medication	Depression
Mood Stabilizer	Lithium 1970	✓		✓	
Atypical Antipsychotics	Aripiprazole 2004	✓	✓	✓	
	Asenapine 2015	✓	✓		
	Cariprazine 2015				
	Lurasidone 2013				✓
	Olanzapine 2000	✓	✓	✓	
	Olanzapine/fluoxetine combination 2012				✓
	Quetiapine 2004	✓			✓
	Risperidone 2003	✓	✓		
	Ziprasidone 2004	✓	✓		
Anticonvulsants	Carbamazepine 2004	✓	✓		
	Lamotrigine 2003			✓	
	Sodium Valproate and Valproate Semisodium1995	✓			
	Lamotrigine 2003			✓	

**Table 2:** NHS Approved Medications For Bipolar Disorder. NHS: National Health Service

Medication Category	Pharmacological Name
Mood Stabilizer	Lithium
Antipsychotic Drugs	Aripiprazole
	Olanzapine
	Quetiapine
	Risperidone
Anticonvulsants	Carbamazepine
	Sodium Valproate
	Lamotrigine

Mode of action of lithium in bipolar disorder

Li is a monovalent (Li^+^) alkali metal and is widely used for the treatment of BD [62]. This alkali metal has been used for almost 60 years for BD as the “gold standard” [63] for mood stabilization [64]. The pathogenesis of BD is complex [6]. Patients with bipolar affective disorder have reduced γ-aminobutyric acid (GABA) neurotransmission, which results in excitatory toxicity. GABA also modulates glutamate and dopamine [65]. Yet to date, the precise pharmacodynamics of lithium regarding BD is not completely elucidated [66]. Nevertheless, recent studies reported that Li persuades various biochemical processes at the cellular level through the modulation of neurotransmission [67,68].

**Figure 2:**
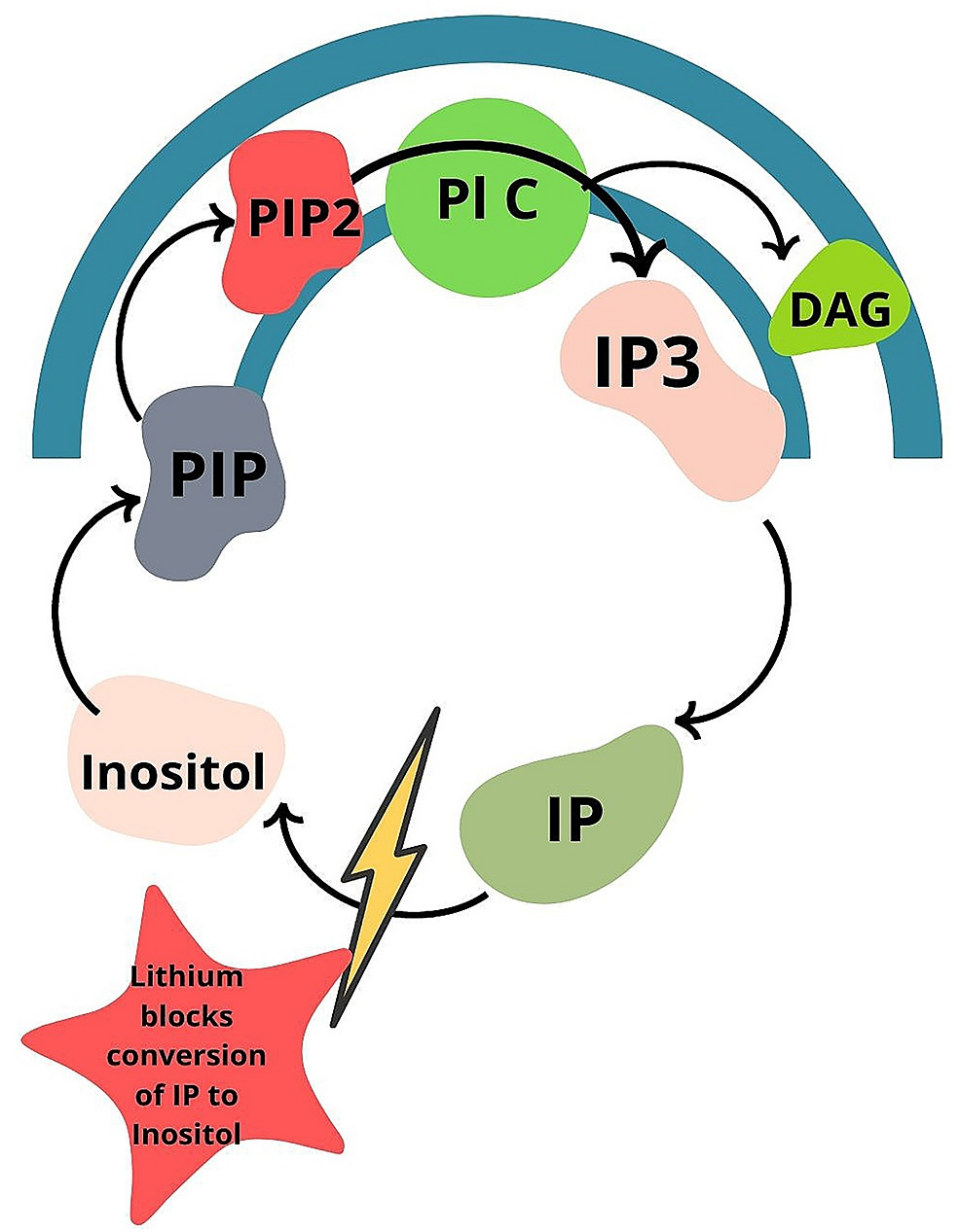
Illustration of Lithium Blocking the Breakdown of Inositol Monophosphate to Inositol. The breakdown of inositol mono phosphate to inositol results in a decrease in free inositol and a subsequent decrease of phosphatidylinositol (PIP2). Image Credit: Rahnuma Ahmad Pl C: Phospholipase C, IP3: Phosphatidyl Inositol triphosphate, DAG: Diacyl Glycerol, IP: Inositol Mono Phosphate, PIP: Phosphatidyl Inositol Phosphate, PIP2: Phosphatidyl Inositol Bis Phosphate.

Li^+^ decreases excitatory neurotransmission at the cellular level by lowering dopamine and glutamate levels. It increases inhibitory transmission by increasing GABA and serotonin levels [66,67]. Li^+^ increases GABA levels, thus directly activating GABA receptors and additionally reducing glutamate and down-regulate N-methyl D-aspartate (NMDA) receptors [66,69]. Li^+ ^alters neurotransmitter and receptor-mediated signaling systems, natural biological forces, hormonal and circadian controlling mechanisms, ion transportation, and gene expression [62,70]. Additionally, Li^+^ targets two enzymatic pathways by inhibiting these enzymes to control BD. One is inositol monophosphatase within the phosphatidylinositol signaling pathway, and the second is the glycogen synthase kinase 3 [65]. Li^+^ affects several second messenger systems. It mainly inhibits the breakdown of inositol monophosphate to inositol; this results in a decrease in free inositol and a subsequent decrease of phosphatidylinositol 4,5-biphosphate (PIP2) (Figure 2). The PIP2 is a precursor of second messengers inositol trisphosphate (IP3) and diacylglycerol (DAG) in the cell membrane. These two-second messengers are responsible for various effects. The PIP2-dependent pathways are thought to be increased in BD, so treatment with lithium is expected to decrease the activity in these pathways [71].

**Figure 3:**
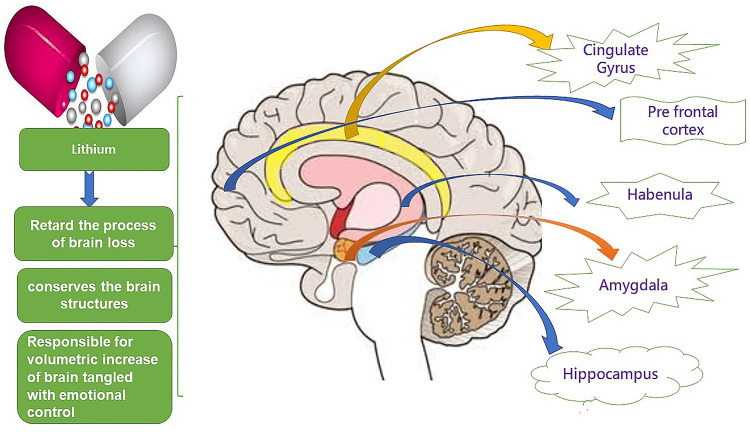
The Effects of Lithium on Brain. This image is depicting the effects of Lithium on the brain including the Prefrontal Cortex, Cingulate Gyrus, Amygdala, Hippocampus, Habenula. Image Credit: Rahnuma Ahmad.

**Figure 4:**
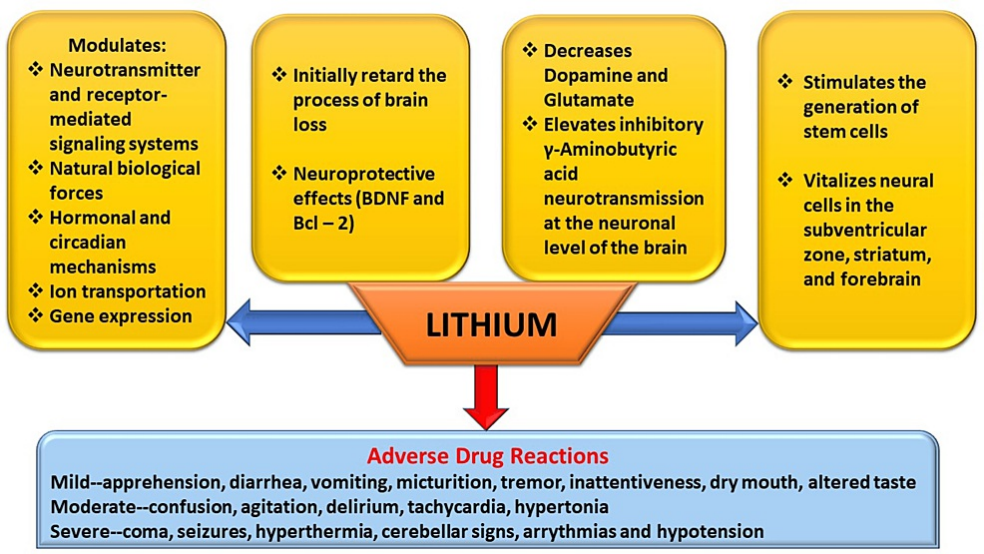
Summary of Effects and Adverse Drug Reactions of Lithium. Image Credit: Siddhartha Dutta. BDNF and Bcl-2 are neuroprotective proteins.

Li initially retards the process of brain loss, primarily responsible for emotional activity among bipolar individuals. It has been reported that lithium possesses not only neuroprotective effects by conserving the brain structures but is also responsible for volumetric increase tangled with emotional control, for instance, the prefrontal cortex, hippocampus, amygdala, anterior cingulate, subgenual anterior cingulate cortex, inferior frontal gyrus, postcentral gyrus, and habenula (Figure 3) [72-74]. Li correspondingly kindles the generation of stem cells, including bone marrow and neural cells in the subventricular zone, striatum, and forebrain. The encouragement of natural neuronal stem cell formation suggests that Li^ ^increases brain tissue mass and capacity among BD individuals [75-77]. At the clinical level, Li is used to control mania; it prevents relapse, reduces suicidal drive in bipolar and unipolar depression, and treats bipolar depression. finally, it prevents bipolar-related cognitive decline at a population level [78,79]. The summary of the effects of lithium is depicted in Figure 4.

Adverse effects of lithium in bipolar disorder

Li^ ^causes mild adverse drug reactions (ADRs) such as apprehension of sickness (feeble, fragile), frequent intestinal evacuations, frequent micturition, deterioration of existing tremor, mild ataxia, dental caries, inattentiveness, poor recall, dry mouth, and an erratic taste in the mouth [80-82]. Moderate ADRs of Li^ ^include confusion, agitation, delirium, tachycardia, and hypertonia. Severe ADRs of Li include coma, seizures, hyperthermia, and hypotension. The serum level of Li for mild, moderate, and severe ADRs were 1.5-2.5, 2.5-3.5, and over 3.5 mEq/L, respectively [83,84]. The effective pharmacotherapeutic range of Li for managing BD is 0.8-1.2 mEq/L [85-87]. 

Lithium vs. antipsychotics/anticonvulsants in bipolar disorder

A systematic review comprising eight real-life studies containing around 14,000 patients revealed that Li^+^ had better clinical results than AC (valproate, lamotrigine, carbamazepine) and atypical AP (olanzapine, quetiapine) [88]. Another study reported that lithium remains the gold standard therapeutic agent for BD despite rising evidence and availability of AC and atypical APs. Nevertheless, it has been recommended that a single medication for the long-standing management of BD is considered ineffective. Multiple agents have been advised for treatment-refractory and rapid-cycling forms of BD [89,90]. However, a study compared different treatment regimens and reported that those patients under Li^+^ + AC (principally lamotrigine and valproate) and Li^+^ + AP (mainly quetiapine and aripiprazole) + AC exhibited a poorer quality clinical outcome than those Li^+^ prescribed as a single agent (*p*<0.01) [91].

**Figure 5:**
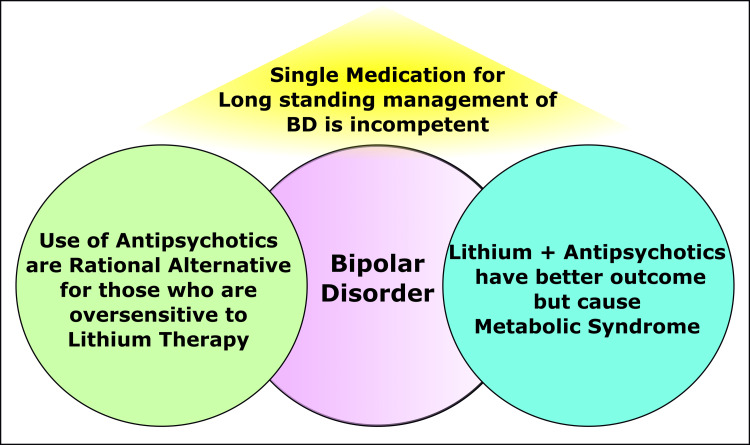
Lithium Versus Antipsychotics in Bipolar Disorder. BD: Bipolar disorder Image Credit: Susmita Sinha.

Additionally, it was shown that Li^+^ + AP is better in managing BD patients, especially in general (*p*=0.05) and manic (*p*=0.01) symptoms than Li^+^ + AC. Unfortunately, ADRs due to APs were noticed to cause metabolic syndrome, especially in glucose and triglycerides [91]. Antipsychotics of the second generation are progressively more prescribed as a single agent or parallelly in combination with other medication for maintenance therapy in BD [92,93]. These newer antipsychotics are considered a single class. Nonetheless, their pharmacokinetics, pharmacodynamics, and ADRs regarding BD have wide-ranging variations [94]. It has been reported that quetiapine, asenapine, and lurasidone show better preventive potential towards depressive episodes of BD [95-99]. Additionally, antipsychotics are the rational alternative for patients with BD who are oversensitive or have poor compliance to Li therapy for relapse prevention (Figure 5) [99,100].

Cost-effectiveness of therapeutic modalities for bipolar mood disorder

Family-based treatment for BD was more effective than hospitalized treatment [101]. This research also recommended that a community-focused mental health program with Li and psychotherapy was found to be the most economical for the prevention of relapse among BD patients [101]. It has been reported that quetiapine + Li^+^ or divalproex Na^+^ possesses better clinical outcomes over placebo + Li^+^ or divalproex Na+ when considered “probabilistic sensitivity analysis (PSA)” among BD [102,103]. Furthermore, quetiapine + Li^+^ or divalproex Na^+^ regimen demonstrated in minimizing severe manic events (46%), critical depressive occurrences (41%), and hospital stay (44%) than placebo + Li^+^ or divalproex Na^+^ [102]. Thereby, the quetiapine + Li^+ ^or divalproex Na^+^ regime showed more cost-efficient long-term therapeutic intervention among bipolar I diseases [102,104]. One pharmacoeconomic research paper evaluating quetiapine cost-efficiency as an ancillary medication with Li^+^ among British and American BD type I cases for long-term pharmacological intervention found a positive result [105].

Furthermore, for BD type II, cost-efficiency is not similarly robust evidence for maintenance therapy over two years [96]. Another European study reported that quetiapine + Li^+^ or valproate Na^+^ decreases acute manic episodes (54%), related hospital stay (29%), improves the quality-adjusted life year (QALY) by 4%, and reduces financial overhead by 5% in comparison to placebo + Li^+^ or valproate Na^+^ among BD type I [106]. Another pharmacoeconomic study reported that quetiapine was more cost-efficient than olanzapine in the therapeutic intervention among depressive BD [107]. One British study evaluating pharmacoeconomic issues among BD type I with manic, mixed, or hypomanic events reported that the Li^+^+ lamotrigine combination remains the most cost-efficient [108]. This combination (Li^+^+ lamotrigine) also potentially defers relapse or recurrence of manic or hypomanic events among BD type I [109].

Role of psychotherapy in bipolar mood disorder

**Figure 6:**
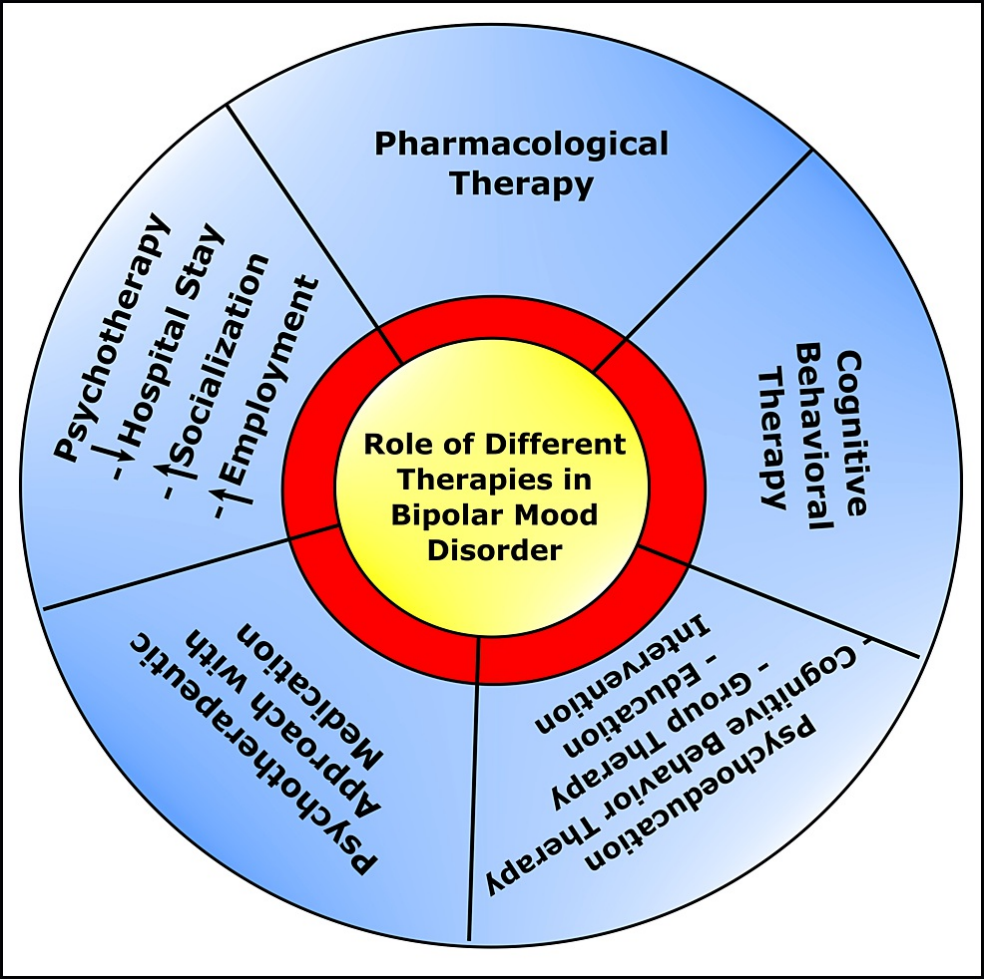
Schematic Diagram Showing Effects of Various Therapies in Bipolar Mood Disorder. Image Credit: Susmita Sinha.

The pharmacological therapeutic options of BD have improved at significant paces [32,110]. Nevertheless, it has been reported that medication alone cannot maintain health-related quality of life (HRQOL) in most cases of BD. These patients need psychotherapy besides drug intervention [111,112]. Long before, psychotherapy was endorsed as an auxiliary therapeutic modality in addition to pharmacological management of BD [113]. Multiple studies reported that the psychotherapeutic approach with close relatives, fellow or kin support, in addition to medication, improves cognitive performances and enhances self-dependency skills among BD cases [11,114-116]. Psychological treatment options, especially cognitive behavioral therapy, are potentially effective in managing depressive events [117-119]. Several studies revealed that psychoeducation is more helpful among patients with manic symptoms of BD. As these patients learn early features of manic episodes, substantial improvements are achieved in socialization and employment [120-122]. Psychoeducation combines cognitive-behavioral therapy, group therapy, and education intervention [123,124]. It has been reported that as psychotherapies reduce hospital stay and improve socialization and employability skills, the psychological therapeutic approach is considered cost-efficient for BD management and other psychiatric issues (Figure 6) [125-127].

## Conclusions

It is evident that lithium exerts a wide range of therapeutic effects on mood and cognition through a complicated network of actions, including neurotransmission and cellular signaling pathways. Therapeutic strategies are still the major focus of BD therapeutic approaches, but drug therapy alone is insufficient for addressing the problem. Lithium appears harmful in studies of cognition in non-psychiatric people, but it seems effective in BD patients. Additionally, it was observed that a community-focused mental health program using Li^+^ and psychotherapy was extremely cost-effective for the prevention of relapsing in BD. Most instances of BD require psychotherapy because medication alone cannot preserve the health-related quality of life. Cross techniques must be used in research and the most recent technological advances in fields like neuroimaging and genetics, in addition to a thorough evaluation of patients with BD. Therefore, by combining basic science research and clinical studies, a more complete picture of the actions of this enigmatic element, Lithium, will spring up.
